# Inorganic and Organic Solution-Processed Thin Film Devices

**DOI:** 10.1007/s40820-016-0106-4

**Published:** 2016-09-08

**Authors:** Morteza Eslamian

**Affiliations:** 1 Photovoltaics Lab, University of Michigan-Shanghai Jiao Tong University Joint Institute, Shanghai Jiao Tong University, Shanghai, 200240 China; 2 State Key Lab of Composite Materials, School of Materials Science and Engineering, Shanghai Jiao Tong University, Shanghai, 200240 China

**Keywords:** Organic electronics, Photovoltaics, Thin film transistors, Thermoelectric devices, Organic light-emitting diodes, Smart materials, Sensors and actuators, Solution-processed methods

## Abstract

Thin films and thin film devices have a ubiquitous presence in numerous conventional and emerging technologies. This is because of the recent advances in nanotechnology, the development of functional and smart materials, conducting polymers, molecular semiconductors, carbon nanotubes, and graphene, and the employment of unique properties of thin films and ultrathin films, such as high surface area, controlled nanostructure for effective charge transfer, and special physical and chemical properties, to develop new thin film devices. This paper is therefore intended to provide a concise critical review and research directions on most thin film devices, including thin film transistors, data storage memory, solar cells, organic light-emitting diodes, thermoelectric devices, smart materials, sensors, and actuators. The thin film devices may consist of organic, inorganic, and composite thin layers, and share similar functionality, properties, and fabrication routes. Therefore, due to the multidisciplinary nature of thin film devices, knowledge and advances already made in one area may be applicable to other similar areas. Owing to the importance of developing low-cost, scalable, and vacuum-free fabrication routes, this paper focuses on thin film devices that may be processed and deposited from solution.

## Background

A thin solid film usually refers to a layer of material ranging from few nanometers to several micrometers in thickness. Thin solid films may be divided into two categories of passive and active films. Passive thin films including coatings are used for aesthetic and decoration purposes, or protection of the underlying surfaces, against moisture, oxygen, high temperature, and mechanical forces to avoid corrosion, surface damage, etc. On the other hand, active thin films can in fact respond to specific triggering effects, such as light, heat, and contact with gases and biological analytes and generate a response for energy conversion, sensing, mechanical actuation, etc. Therefore, the combination of one or more active thin films, and perhaps some passive thin films, can make a thin film device, such as thin film solar cells (SCs), transistors, thermoelectric devices, sensors, and actuators, to name a few. Various types of thin film devices may share similar principles of operation or fabrication processes, and therefore advances in one device may provide new windows of opportunity for the development of other devices. As an example, molecular semiconductors, such as perovskites developed for perovskite SCs, may be used in other devices, such as thin film transistors. Or most of the thin film devices employ graphene and carbon nanotubes in their structures, and advances in one field may be utilized in other fields, as well.

**Fig. 1:**
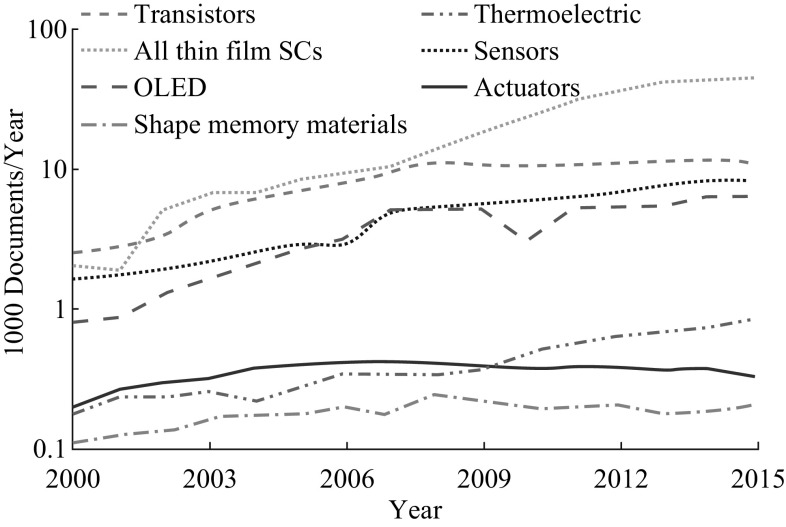
Number of scientific publications found in the Web of Science on topics of thin film transistors, thin film thermoelectric devices, thin film solar cells (SCs), thin film sensors, organic light-emitting diodes (OLEDs), thin film shape memory materials, and thin film actuators, within the timespan of 2000–2015

In this paper, the following topics will be considered. First, the methods of materials processing and thin film deposition will be discussed with emphasis on colloidal and solution-processed methods, suitable for scalability and commercialization of thin film devices. This is because deposition methods will be frequently referred to in the subsequent sections. Then the principles of operation and recent advances in thin film transistors, memory for data storage, inorganic and organic light-emitting diodes (OLEDs), organic and inorganic thin film SCs, thermoelectric devices, chemiresistive sensors, mechanical actuators, and shape memory materials (SMMs) will be reviewed, to provide insight for future interdisciplinary research in thin film devices. To highlight the relative impact and the trend in each field, the number of scientific publications on various thin film devices found in the Web of Science within the timespan of 2000–2015 is shown in Fig. 1. The y-axis is in the logarithmic scale. It is shown that the research in thin film devices is still dominated by thin film SCs and then in the order of abundance by thin film transistors, sensors, and OLED. The figure however shows that research in thin film SCs is slowing down or saturating, mainly due to a drop in the cost of polysilicon used in high-efficiency silicon SCs, which has challenged the feasibility of inorganic and emerging SCs.

## Solution-Processed Thin Films

A thin solid film may be deposited from precursors in vapor, liquid, or even solid phases, or a combination of several phases, depending on the nature of the precursors and the desired functionality and specification of the resulting thin solid film. Physical and chemical vapor deposition methods, such as sputtering and epitaxy, are some examples of vapor phase deposition methods. These processes require well-controlled atmosphere and are usually performed in vacuum, using expensive equipment and energy-intensive processes. Therefore, it is not surprising that the resulting thin solid films made using vapor phase methods are usually of high quality in terms of the micro- and nanostructures of the films and the low density of defects. On the other hand, the liquid-phase methods for deposition of thin films from solution or colloidal mixtures, such as printing methods, are cheaper but less controllable and repeatable, which may affect the quality of the resulting thin solid films. It is noted that some thin films may be deposited from several methods, whereas some other are only compatible with a particular deposition route. For instance, highly crystalline thin films of semiconductors need to be deposited via the vapor phase methods, such as epitaxy, or organic thin films are usually deposited from a low-temperature solution, whereas some other materials, such as copper indium gallium selenide (CIGS) inorganic thin film semiconductors, may be deposited from both the vapor and liquid phases. The vapor phase film deposition methods are well studied and established. Therefore, the focus of this paper is more on potential for large-scale fabrication of thin film devices that can be processed in solution using wet chemistry and deposited using proper printing or coating techniques.

In chemical vapor deposition route, the deposition process and the chemical reactions to convert the precursors to the desired thin film material occur concurrently. Physical vapor deposition route, in contrast, only involves the deposition of vaporized precursors from the gas phase onto the substrate. In solution-processed deposition methods, a very similar scenario repeats, i.e., solution-processed methods may be physical or chemical. In physical solution-processed methods, the liquid precursor, in the form of a colloidal ink or a solution, is simply cast onto a substrate ideally forming a thin liquid film that is subsequently dried and possibly sintered to form a thin solid film. In chemical-based solution-processed methods, the mixed precursors undergo a chemical reaction, such as sol–gel, solvothermal, and pyrolysis, before, during, or after the casting stage. The chemical reactions and casting may proceed simultaneously, such as that in spray pyrolysis. The main casting methods that can be used to deposit thin films include but are not limited to drop-casting, spin coating, blading (knife-over-edge), gravure, slot-die, screen printing, inkjet printing, and spray coating [1–4]. Each method has its advantages and disadvantages and may be more suitable for a particular application. Solution-processed methods require the application of effective solvents and surfactants to control the solubility, surface tension, viscosity, and other physical and chemical properties of the solution to achieve a desired thin film. This is quite challenging and requires optimization process and experience to develop a perfect recipe that can generate the desired thin films.

As mentioned above, in solution-processed deposition methods, first a thin liquid film is cast, at once such as that in spin coting, or drop by drop such as that in spray coating and inkjet printing, followed by subsequent heating, depletion of solvents and additives, and possible chemical reactions, to finally obtain a thin solid film. Study of thin liquid films requires understanding of complex fluid mechanics, thermodynamics, and surface science phenomena, such as wetting/dewetting, formation of surface waves and film instability and breakup, pattern formation, evaporation, interaction between the film interfaces in ultrathin films, the effect of external forces, such as centrifugal forces in spin coating, on the behavior of the film, etc. This makes the field of solution-processed thin films and thin film devices a multidisciplinary field that entails in-depth knowledge of wet chemistry, fundamentals of hydrodynamics, thermodynamics, and surface and interface science associated with thin liquid films, nanostructure and physical and chemical properties of thin solid films, and principle of operation of the desired thin film device.

Each deposition technique mentioned above is governed by a set of physical laws, specific to that method only. For instance, in lab-scale and for research and development purposes, most thin film devices are fabricated by spin coating; however, the knowledge and experience gained during device fabrication by spin coating stays in the lab only, because scalable and commercially viable techniques, such as slot-die coating, inkjet printing, and spray coating, are governed by different sets of physical laws. Spin coating is a controllable technique governed by a rather simple hydrodynamic equation and capable of the fabrication of thin films with any thickness from few nanometers to micrometers by adjusting the solution concentration, angular velocity, and spinning duration [5]. The wet spun-on films may still dewet due to the growth of perturbations in metastable liquid films, but overall the process is easy to control. Drop-casting is another simple lab-scale deposition method for the fabrication of small-area thin films. An impinging drop spreads on the substrate because of its momentum and may wet the surface if the surface has high energy or the contact angle is low. Therefore, the film uniformity and thickness highly depend on the momentum and physical properties of the impinging drop (Reynolds and Weber numbers), as well as the substrate wettability and texture. Drop-casting may become more controllable by applying external forces, such as imposing ultrasonic vibration on the substrate [6]. Slot-die printing is a non-contact method and works based on continuous release of the precursor solution from a moving die above a substrate to form a liquid film, which subsequently dries to form a solid film. Its principle of operation is simple and the process is controllable; however, the method is not suitable for deposition of ultrathin films, because a very thin liquid film may break up during liquid transfer from the die to the substrate. This method has been successfully deployed for the fabrication of some layers of organic SCs incorporating thin solid films of about 1 μm or so [4]. Blade coating or knife-over-edge coating is another method which has been used in small scale, as well as larger scale, for making thin film devices [4]. In blade coating, the blade translates in close proximity or in contact with the substrate. Therefore, the roughness, uniformity, and flatness of the substrate or the underlying film and the gap between the blade and the substrate determine the quality and thickness of the deposited film. Inkjet printing relies on controlled ejection of many ink droplets from a piezoelectric transducer onto the substrate. Ink is a colloidal mixture or a solution of the precursors. Droplet impingement, spreading, and coalescence of such droplets result in the formation of wet lines and films and thin solid films after solvent evaporation. Inkjet printing is much more controllable and reproducible compared to spray coating, although the former is a low-throughout casting method compared to the latter. Spray coating, although fast and low-cost, is inherently a stochastic and random process in microscale, involving complex droplet inflight and impact dynamic processes [7]. Obtaining smooth and uniform spray-on thin films of some precursor solutions is therefore challenging. In addition, application of masks may be needed to obtain spray-on thin films with specific dimensions and patterns. Both inkjet printing [8, 9] and spray coating [10–14] have been extensively used for the fabrication of a variety of thin film devices in bench and pilot scale, although the results are difficult to reproduce by others. The conventional approach to tune the nanostructure of solution-processed thin films is via solvent treatment, extensively performed to improve the device performance [15, 16]. This is, however, a tedious and expensive process with adverse environmental footprints. A mechanical treatment has been recently developed in which a forced ultrasonic vibration is imposed on the substrate to enhance merging of droplets and mixing of precursors to level the film surface and to improve the film nanostructure [17, 18]. The imposed vibration has also worked well on wet films cast by spin coating, resulting in improved functionality of organic thin films [19]. Therefore, it is deduced that the application of other contact or non-contact external forces may improve the film uniformity and nanostructure. The use of electrosprays or electrostatic sprays, in which a high voltage applied between the nozzle tip and the substrate controls the atomization process, is another means to manipulate the atomization process in sprays and thus control the quality of the deposit [20]. A limitation of inkjet printing and spray coating is that since the aforementioned methods rely on droplet and spray generation from the emergence of liquid from a small capillary, the precursor solution or ink should have favorable physical and chemical properties to allow successful discharge and breakup of the liquid without precipitation of solute or colloidal particles in the pipelines or nozzle. Therefore, the development of inks with favorable properties employing very small colloidal nanoparticles and also the development of novel droplet generators and atomizers that can generate a uniform spray may further advance this area.

In most thin film devices, the device comprises several stacked layers of thin films that serve to fulfill various functionalities. This requires deposition of several thin films atop one another, imposing additional challenges. First of all, the solvent used in the precursor solution to prepare and deposit a layer atop an existing layer should be chosen properly to avoid dissolution of the underlying layer. Also, the deposition should be controlled precisely to prevent the formation of voids between the interfaces of the two films. Existence of voids results in excessive resistance between adjacent layers affecting the device performance. Therefore, much effort is required to engineer the interfaces, as well as each individual layer.

In contrast to usually precise vapor phase vacuum-based deposition methods, in which the film forms gradually and even atom by atom, in solution-processed methods, the film is deposited usually at once in the bulk form, limiting precise control over the film nanostructure. If intermolecular forces, which shape the nanostructure and crystallinity of the film, are small, such as in organic materials, the crystallinity, grain size, and crystal alignment of the resulting thin solid films will be sensitive to the fabrication conditions, such as solvent evaporation rate, solvent type, imposed forces, etc. Overall, although the application of solution-processed deposition methods has attracted enormous attention in academia and industry to fabricate thin film devices, the lack of sufficient controllability and reproducibility in micro- and nanoscale and low device efficiency or instability are obstacles in the way of commercialization of such technologies, evidenced by bankruptcy of some small and large companies in this field, in particular in organic photovoltaic industry [21]. Some of the shortcomings will be overcome in the future by performing in-depth fundamental studies in all aspects of solution-processed thin film devices. Currently, at least in the lab scale, the solution-processed method is an important and versatile route for the preparation and deposition of organic thin film devices and some inorganic thin film devices with moderate crystallinity. It has been shown that quantized energy levels in ultrathin layers of oxides, such as ZnO, processed from solution can form, by tuning the solution concentration and deposition parameters [22].

**Fig. 2:**
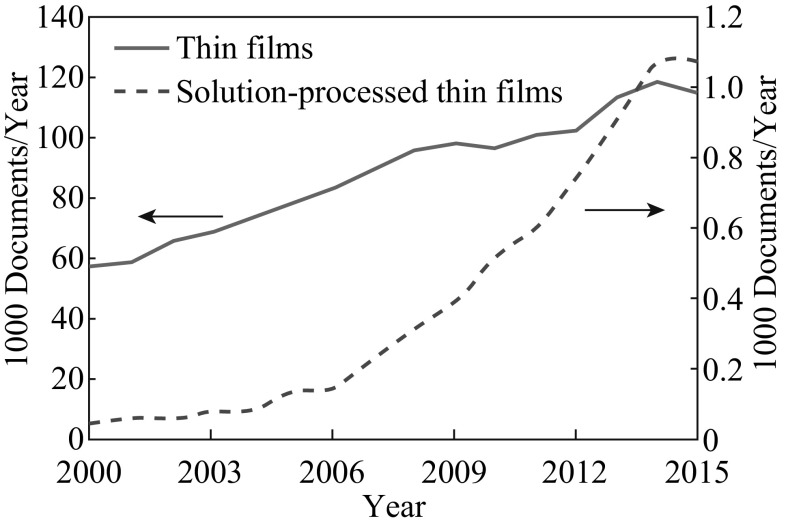
Number of scientific publications found in the Web of Science on “thin films” and “solution-processed thin films” or “solution-based thin films” within the timespan of 2000–2015

To substantiate the importance of the solution-processed deposition methods, the number of scientific documents found in the Web of Science on “thin films” and “solution-processed or solution-based thin films” within the timespan of 2000–2015 are compared in Fig. 2. It is deduced that the number of documents reporting the application of solution-processed methods in thin films is rapidly increasing with a positive rate of change. In the following sections, emerging organic and inorganic thin film devices are reviewed, with emphasis on devices that can be fabricated using solution-processed casting methods.

## Thin Film Transistors

**Fig. 3:**
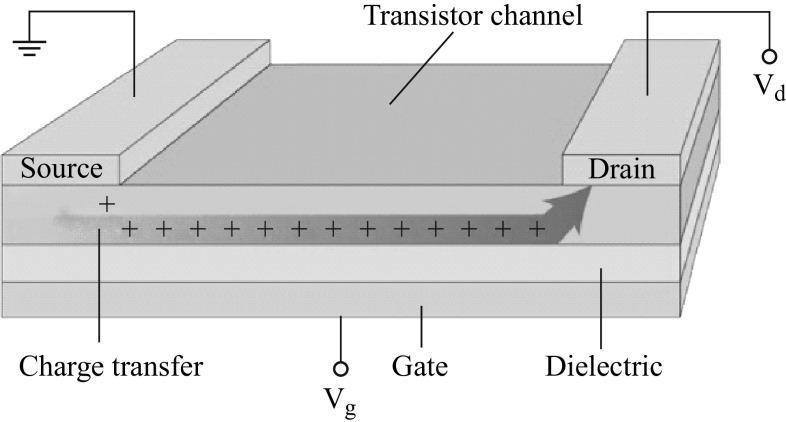
Schematic of an organic field-effect transistor (OFET). Heavily doped silicon is a traditional substrate, while silicon dioxide or other dielectrics serve as the gate insulator

A transistor is a device usually used to amplify or switch electronic signals. There are two types of transistors: bipolar and unipolar (field-effect transistor, FET). A FET has three terminals: source, drain, and gate. A FET usually operates as a capacitor and its internal body is composed of two plates. One plate is a semiconducting inorganic or organic conducting channel devised between two ohmic contacts: the source and the drain. The other plate (gate) controls the charge induced into the channel. Charge is induced from source to drain, only if the gate voltage is higher than a threshold value. Based on this concept, the FET can have several configurations, including the thin film configuration, the focus of this paper. In thin film transistors (TFTs), doped silicon, indium tin oxide (ITO), or other conducting materials are used as the gate, a dielectric serves as the gate insulator or capacitor, and a semiconductor, such as conducting polymers or metal oxides, serves as the channel (Fig. 3).

The currently used and ideal conducting channel in TFTs is microcrystalline silicon with high mobility. However, in recent years interest in organic semiconductors [23] and solution-processed inorganic semiconductors, such as oxides [24], has grown, because such materials may be processed and deposited from solution, providing the opportunity to reduce the fabrication cost [25]. Organic semiconductors may be readily processed in solution. Also, some metal oxides are relatively insensitive to the presence of structural disorder typically associated with solution processing, and therefore high charge carrier mobility is achievable in such inorganic structures [26]. Issues such as improving the transparency, charge mobility, stability and developing reproducible and scalable solution-processed deposition methods need to be addressed to pave the way for commercialization of the low-cost solution-processed TFTs for application in flat-panel display backplane, flexible displays, sensor arrays, etc. For instance, the carrier mobility, which is an important figure of merit of transistors, is affected by the film crystallinity and the nanostructure, but the solution-processed materials usually have a low mobility due to the presence of imperfections in the film nanostructure. To improve the nanostructure and therefore mobility, using an off-center spin coating method to align the polymer chains in one direction, Yuan et al. [27] fabricated a transparent thin film organic FET (OFET), with a high mobility of 43 cm^2^ (Vs)^−1^. To take advantage of the external centrifugal force to align the conducting polymer chains to create charge carrier pathways, the substrate was mounted at a distance away from the axis of rotation. The idea of using external forces to align the polymer chains and therefore increase the conductivity has been employed by others as well, such as imposing ultrasonic vibration on wet films [19]. Azarova et al. [28] fabricated spray-on polymer transistors with good homogeneity, but mobility of the channel was rather low (0.2 cm^2^ (Vs)^−1^). Application of spray coating for deposition of narrow transistor channels requires the employment of masks to deposit targeted areas only. In line with the scaling up of the technology, flexible and light TFTs and circuits were fabricated by Fukuda et al. [29] for wearable and foldable displays and medical applications using transfer printing method processed from solution. In most solution-processed devices, the electrodes are usually deposited by thermal evaporation or sputtering. To achieve a fully solution-processed OFET, for scalability and commercialization purposes, silver nanoparticles were deposited from solution and sintered to fabricate the electrodes [30]. This is applicable to all thin film devices; however, in this approach, achieving suitable ohmic contact between the electrode and the underlying layer is a challenge. Development of new conducting thin films, such as thin films incorporating graphene as a transparent electrode, is another research direction in this field.

The advantage of inorganic semiconductors over organic counterparts is their stability against degradation. While organic materials are usually flexible, transparent, and solution-processed at low temperatures, they are prone to degradation, when exposed to heat, moisture, oxygen, etc. As an example of solution-processed oxide TFTs, Yu et al. [31] used composite In_2_O_3_ thin film doped with an insulating polymer poly (4-vinylphenol) (PVP) to fabricate a transparent and flexible TFT. PVP was selected due to its excellent solubility in the In_2_O_3_ precursor solution. The device was fabricated on flexible amorphous Zn_0.3_In_1.4_Sn_0.3_O_3_ (ZITO)-coated AryLite polyester substrate, exhibiting 80 % transparency. Solution-grown amorphous alumina (AlO_*x*_) was used as the dielectric and ZITO as the source and drain electrodes. The device showed a mobility of about 1.5–3.2 cm^2^ (Vs)^−1^, depending on the process temperature and concentration of PVP in the metal oxide. Banger et al. [26] fabricated a TFT, based on amorphous metal oxide semiconductors, using the sol–gel processing and spin coating, where a mobility of 10 cm^2^ (Vs)^−1^ was achieved. Their device was composed of glass, heavily doped silicon as the gate, silicon oxide as the dielectric, and indium zinc oxide and indium gallium zinc oxide as the channel semiconductors. Using ultrasonic spray pyrolysis, Petti et al. [11] fabricated TFTs with indium oxide as the conducting channel with a mobility of up to 16 cm^2^ (Vs)^−1^. The TFTs were fabricated on doped silicon or flexible polyimide foil. In an attempt for low-cost fabrication of thin film transistors for flexible electronics, Lau et al. [32] reported fully printed transistors using inverse gravure printing technique. The TFTs were configured in the top-gate device geometry and utilized semiconductor-enriched carbon nanotubes as the active channel material, silver metal electrodes, and an inorganic/organic gate dielectric. The devices exhibited mobility and on/off current ratio of up to 9 cm^2^ (Vs)^−1^ and 10^5^, respectively.

In addition to the existing conducting polymers and inorganic materials, the application or synthesis of new advanced functional materials is an important research direction. Irimia-Vladu et al. [33] investigated the application of several biodegradable materials, such as sugar family and food dye and biodegradable polymers as layers of OFETs, to address the issue of plastic waste disposal in plastic and polymer electronics. In addition, the application of such biodegradable materials may provide future opportunities to fabricate micro- and nanodevices to be used inside the human body for diagnostic or treatment purposes.

The forgoing literature review and discussion reveals the potential of solution-processed thin film transistors for emerging applications, such as wearable devices and foldable and transparent sensors, displays, and medical devices, although these technologies are still in their infancy. In this field, some of the research directions include the development of stable semiconducting organic and inorganic materials with high mobility and on–off ratio for the conducting channel and exploring scalable solution-processed methods. For instance, some of the molecular semiconductors developed for other thin film devices, such as lead halide perovskites, may work well also in thin film transistors. Theoretically, perovskite can achieve charge mobility higher than that of crystalline silicon [34].

**Fig. 4:**
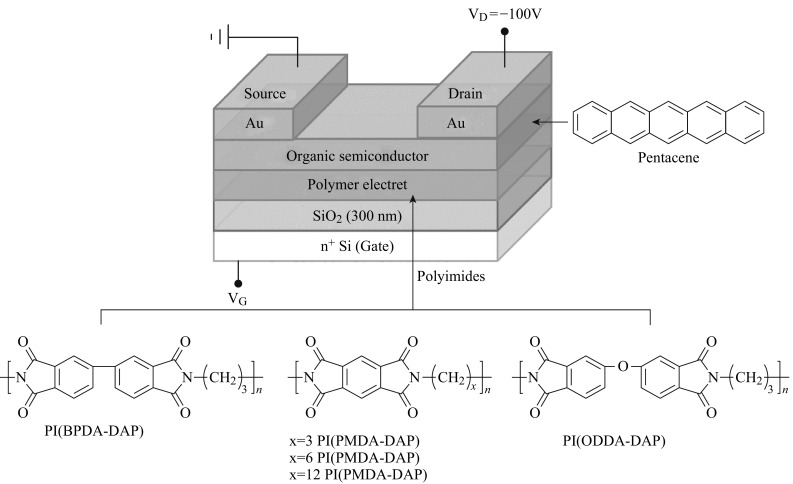
Structure of an organic non-volatile memory transistor with a polymer electret, based on semi-conjugated acceptor-based polyimides [37]

In addition to signal switching and amplification, transistors are used in memory devices for data storage, as well. The advanced data storage technologies include the semiconducting, magnetic, and optical methods. Semiconductor memories have much faster access times than other types of data storage technologies. A semiconductor memory chip may contain millions of transistors and capacitors for volatile or non-volatile data storage. As far as the thin film technology is concerned, the two ongoing research directions in memory devices include the solid-state semiconductor technology for non-volatile memories [35] and molecular memories using thin film organic transistors [36]. Organic memory devices are solution-processed and compatible with integrated circuits composed of other organic electronic components. Compared to the regular TFTs, transistor memories have an additional charge storage layer between the gate contact and the organic semiconducting channel. Every memory cell contains one transistor allowing storage of at least one bit through an additional electric field (voltage bias or pulse) applied to the gate contact. The charge can be stored within the bulk of the dielectric film or at the interface between the gate dielectric and the channel. Figure 4 shows a polymer electret-based transistor memory, a kind of dielectric material that exhibits a quasi-permanent electric field caused by trapping of the electrostatic charges [37]. The utilization of a polymeric charge storage electret as a reversible charging dielectric is a simple and effective strategy toward high-performance non-volatile organic thin film memories. Various types of polymers and polymer composites that have the charge storage capability have been developed and tested, such as pendent polymers, donor–acceptor polyimides, and polymer composites [36]. The memory functionalities resulting from the channel conductance modulation by trapped charges inside the polymer electret upon applying the programing or erasing gate voltage and the charge storage capability is extensively dependent on the composition, nanostructure, and chemical nature of the electrets. The current setback in molecular memory devices is the low retention time, which is the ability of the transistor memory device to preserve the programming/erasing states without losing the trapped charges due to leakage over time. Also, controlling the process parameters, which are currently difficult to achieve in solution-processed methods is another hurdle in scaling up and commercialization of this emerging technology.

Another reported application of OFETs is their ability to sense force and pressure in a quantitative manner [38]. The transistor sensor is composed of pentacene as the conducting channel, and solution-processed polyvinylphenol as the dielectric, deposited on glass substrates. Application of uniaxial mechanical pressure with a needle resulted in response of the transistor. The mobility, switch-on voltage, and interface resistance were affected by the applied pressure, and thus the transistor could be used as a pressure sensor. The origin of the observed effect was attributed to trapped charges, as a result of mechanical pressure.

## Organic Light-Emitting Diodes (OLED)

**Fig. 5:**
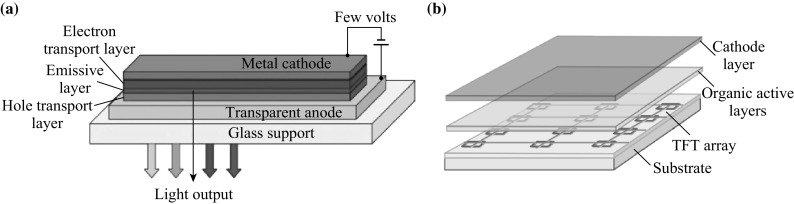
**a** Schematic of various layers of a thin film organic light-emitting diode (OLED) and **b** the structure of the commercialized active-matrix OLED display (AMOLED) technology used in smartphones

Incandescent, fluorescent, and light-emitting diodes (LED) are technologies for converting electricity into light for lighting and display applications. LEDs have the potential to exceed the efficiencies of conventional incandescent and fluorescent devices. LEDs work based on the electroluminescence effect in a semiconductor, in which the P–N junction of a semiconductor emits light, as a result of recombination of opposite charges that reach the junction, when a voltage is applied to the semiconductor. The principle of operation of LED is somewhat the opposite to that of the photovoltaic SCs. With the emergence of conducting and semiconducting polymers, OLEDs, which are similar in operation to LEDs, but use organic semiconductors instead of conventional inorganic semiconductors, have received enormous attention in recent years. The OLEDs are composed of several layers of thin films including hole injection and transport layers, an electron–hole recombination layer (emissive), and an electron injection layer. These multiple layers are used to facilitate a smooth flow of electrons and holes from the electrodes to the emissive layer. These layers are sandwiched between two electrodes, one of which is transparent. In an organic semiconductor, the mobility of electrons is much lower than that of the holes, making it difficult to balance the system. Over many years of research and development, various florescent, photofluorescent, and hybrid materials have been developed for the emitter layer to achieve high quantum efficiencies. Discussion of characteristics of these materials is beyond the scope of this paper and may be found in several reviews in the field of OLEDs, e.g., [39–41]. Figure 5a shows the basic schematic of an OLED and Fig. 5b shows the AMOLED (active-matrix organic light-emitting diode) structure, which is the commercial version of the OLED circuit, used in a variety of electronic devices with a display, such as smartwatches, mobile devices, laptops, and televisions. An AMOLED display consists of an active matrix of OLED pixels, deposited or integrated onto a thin film transistor (TFT) array, where the TFT controls the current flowing to each individual OLED pixel. The TFT backplane technology is crucial for the fabrication of AMOLED displays. In AMOLEDs, the two primary TFT backplane technologies, polycrystalline silicon (poly-Si) and amorphous silicon (a-Si), are currently used to offer the potential for directly fabricating the active-matrix backplanes at low temperatures (below 150 °C) onto flexible plastic substrates for producing flexible AMOLED displays. With further development of organic thin film transistors (OTFTs), the fabrication process can be further simplified, making all-organic displays, i.e., OTFT + OLED, using solution-processed deposition methods.

LEDs and OLEDs can be used for both lighting and electronic displays. OLED lighting panels have reached high efficiencies and are available in the market, although the price is still high, and therefore are used for decoration. OLED displays have been commercialized as well and can be found in the market in flat-panel televisions, laptops, cellular phones, etc. Contemporary displays are of two kinds: liquid crystal display (LCD), which is the dominant type, and the emerging OLED displays. The LCD consists of a panel of liquid crystal molecules that can be induced by electrical fields to take certain patterns which block light or allow it through. However, the crystals create no light of their own [42]. Current LCD technology uses LEDs to backlight the LCD panel, while OLED pixels produce their own light individually and OLED displays are thinner, more energy efficient, and have a shorter response time, but are costly. The white-light OLED technology has already been successfully deployed in a series of OLED televisions by LG Electronics. Samsung previously manufactured red–green–blue (RGB) OLED televisions, but it was discontinued because of the high cost of manufacturing of defect-free RGB-based OLED TVs, and concerns associated with the lifetime, given that organic molecules are prone to degradation over a relatively short period of time, resulting in color shifts as one color fades faster than another [43].

OLED devices and displays are currently fabricated by thermal evaporation using shadow masks [44]. Thermal evaporation is energy intensive but results in uniform, defect-free, and dense layers, although a large amount of material is wasted in the chamber. It is generally a sheet-to-sheet process, but can become roll-to-roll compatible, if the whole process is performed in a large coating chamber. Solution-processed fabrication of OLEDs is the future technology, which is currently under research and development. As discussed in previous sections, the solution-processed approaches, such as coatings and printing, are cost effective and suitable for roll–roll fabrication of large areas at ambient condition, but suffer from the lack of process controllability and difficulties associated with the preparation and treatment of solutions to achieve defect-free large-area films. In 2010, DuPont revealed that they could produce a 50-inch OLED TV in 2 min with a new multi-nozzle spraying technology. If scaled up, then the total cost of OLED TVs would be greatly reduced. DuPont also stated that OLED TVs made with this technology could last up to 15 years, if left on for a normal 8-h day [45].

Some challenges associated with the OLEDs include the need for improvement of the electricity-to-light conversion efficiency by using and synthesizing new materials, improvement of the stability and lifetime of the organic materials, developing stretchable and foldable displays [46], and developing proper encapsulation methods and low-cost solution-processed approaches, while the film uniformity over large areas can be maintained. One research direction in this field is the development of colloidal quantum dot LED (QLED), which employs a thin film of quantum dots as its emissive layer, shares many of the advantages of OLED, has the potential for lower cost fabrication using printing technologies, and is expected to become commercialized within few years [47]. Quantum dots are inorganic semiconductors, which are more stable than organic counterparts. Development of inorganic–organic perovskite light-emitting diodes has been reported, as well [48]. In addition to electronic displays and lighting, some other possible applications of OLEDs and solution-processed LEDs include but are not limited to touch-sensitive textile displays, personal healthcare and monitoring devices, continuous treatment management devices, environmental detection of contaminants and pathogens, and explosives detection, with either a wearable or a low-cost one-time use application [49].

## Thin Film Solar Cells

Solar energy is a rather democratic resource of renewable energy available to all countries and regions, literally for free. Conversion of solar energy directly to electricity through the photovoltaic (PV) effect is the ideal way of utilization of this abundant energy. The conventional use of solar energy for power generation via the solar thermal (concentrated solar power) is less efficient given that the solar energy is first converted to heat for steam generation in a boiler and then electricity is generated in a thermodynamic cycle, which is restricted by the second law of thermodynamics. A PV SC works based on the absorption of light, excitation of electrons in a semiconductor, generation of electron–hole pairs, separation of charge carriers of opposite types, and collection of charge carriers at opposite electrodes. Therefore, a SC is composed of an active layer or light absorber, as the heart of the SC, and several buffer layers to control the selective direction of flow of the charges, all sandwiched between two electrodes. The first-generation PV SCs are based on crystalline silicon, which comprises rather thick (several hundreds of micrometer) silicon films, due to the limited absorption coefficient of silicon. To save on the materials and fabrication costs, reduce the module weight, and improve the module flexibility, the second-generation SCs, based on inorganic semiconductor thin film concept, have been developed. Cadmium telluride (CdTe), CIGS and other chalcopyrites, copper zinc tin sulfide (CZTS) and other kesterites, and amorphous silicon (a-Si) are of second generation of SCs [50]. In the aforementioned inorganic thin film semiconductors, few nanometers up to few micrometers of the light absorber are sufficient to absorb a large portion of the sunlight. Commercialized inorganic thin film SCs are usually deposited from the vapor phase; however, in an attempt to reduce the cost, research is being undertaken to fabricate such SCs using solution-processed methods [1]. The third generation of SCs, also called emerging SCs, is still in the category of thin film SCs, but most of the layers of such SCs are processed in solution and deposited using a casting method. These include organic (polymeric) [3], dye-sensitized [51], perovskite [52], and quantum dot SCs [53].

**Fig. 6:**
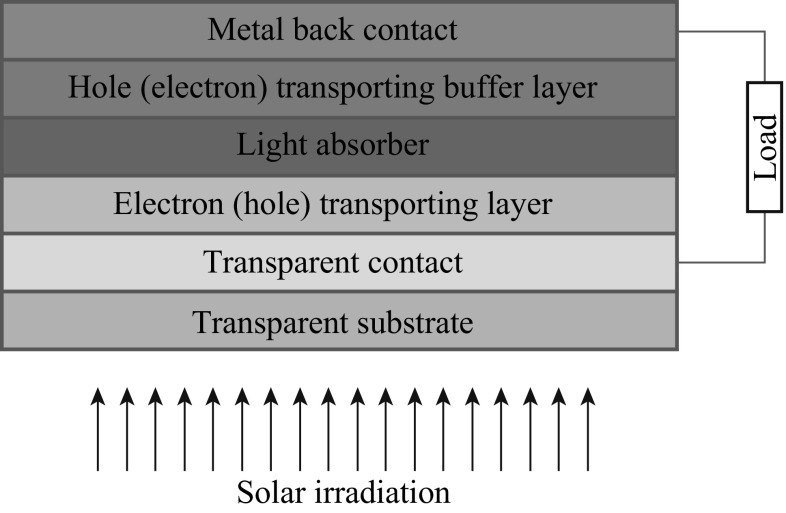
Schematic of the structure of a thin film solar cell

Figure 6 shows the typical structure of a thin film SC, which applies to both second- and third-generation SCs. The active layer or the light absorber varies from one cell to another, and the cell is named based on the semiconductor used as the active layer. In order to facilitate the flow of electrons and holes to opposite electrodes, the buffer layers and electrodes are selected according to their work functions and compatibility with adjacent layers in terms of materials processing and fabrication. A thin film SC in dark or without the active layer is a thin film diode, allowing the charge transfer in one direction only.

Based on the latest report by the National Center for Photovoltaics (NCPV) at the National Renewable Energy Laboratory (NREL) of the United States of America [54], the highest power conversion efficiencies (PCEs) of some of the thin film SCs, with the capability to be made using low-cost solution-processed methods, are as follows: organic or polymeric SCs—11.3 %, dye-sensitized SCs—11.9 %, perovskite SCs—22.1 % (not-stabilized), quantum dot SCs—11.3 %, inorganic chalcopyrite CIGS SCs—22.3 %, and inorganic kesterite CZTSSe SCs—12.6 %. The efficiency chart shows that among the solution-processed SCs, the record PCE of the dye-sensitized SCs has not increased in the past few years. Perovskite SCs have reached a PCE of 22.1 % within few years, although the perovskite structure tends to degrade, when exposed to light, heat, moisture, oxygen, etc. [52]. The improvement in the performance of polymer SCs has also slowed down, significantly. Quantum dot SCs are still progressing with a slow, but consistent pace.

The PCE reported for SCs can be quite misleading, when it comes to their practical applications. The PCE is a physical figure of merit of the cell, as far as the effectiveness of the cell in terms of the conversion of solar energy to electricity is concerned. However, given that the fuel which is the incident solar energy to the SC is free, the more meaningful efficiency of the cell, to compare a SC with other SCs, and with other sources of energy, is the ratio of the total cost of the fabrication and installation per unit of the maximum energy or power that can be generated under standard test conditions, i.e., ($/Wp) or $/kWh, respectively. Utility-scale solar power based on silicon technology can now be delivered in California at prices below $0.10/kWh, less than most of other peak generators, even those running on low-cost natural gas [55]. This is owing to the advances made in crystalline silicon technology, in recent years, and the falling cost of the polysilicon feedstock in 2009, followed after a period of global shortage. This has pressured the manufacturers of commercial thin film technologies, including a-Si, CdTe, and CIGS SCs. In the past 20 years, the Chinese manufactures have continuously increased their capacity in the market, leading to a drop in the cost of silicon solar panels. As of 2013, silicon technology is accounted for over 90 % of the PV market and the remainder is taken by a-Si, CdTe, and CIGS thin film SCs [56]. Thin film SC manufacturers continue to face price competition from the Chinese producers of silicon and manufacturers of conventional crystalline silicon solar panels. However, after a period of continuous and rapid decline in the price of silicon solar panels, the manufacturing costs seem to have approached a steady value. The total energy price comprises the module cost, as well as the administrative, marketing, labor, installation, and the balance of system costs, some of which cannot be reduced any further. Therefore, no further major reduction in the cost of installed silicon solar panels is expected. This may increase the efforts and investments to further develop and commercialize inorganic and emerging SCs at lower costs. On the other hand, given that in recent years the thin film inorganic and emerging solar technologies have lost ground and feasibility, more focus can be placed on unique characteristics of these technologies, such as light weight, flexibility, and transparency, to develop SCs to be used in wearables, portable devices, solar windows, curtains, walls, etc.

**Fig. 7:**
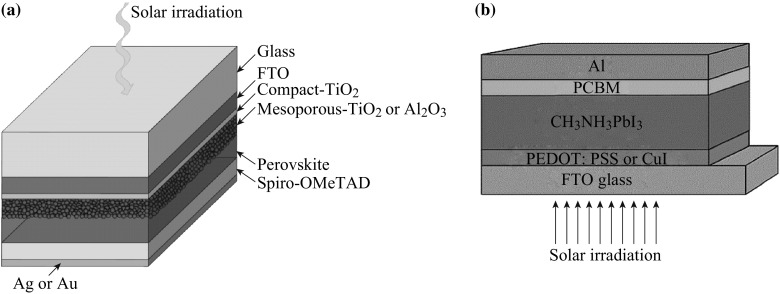
**a** Structure of a mesoporous perovskite SC, where FTO is used as the transparent conducting electrode, c-TiO_2_ as the ETL, and spiro-OMeTAD as the HTL. A planar structure is similar to (**a**), except that it does not have the mesoporous scaffold. **b** Structure of an inverted planar perovskite SC

Among emerging SCs, perovskite SCs are more promising than quantum dot, polymer, and dye-sensitized SCs. In the category of inorganic thin film SCs, CIGS and CZTSSe SCs have acceptable PCEs and stability and can be processed in solution and deposited using coating and printing methods, as well as the conventional evaporation-based methods. Thus, here advances and opportunities regarding the aforementioned promising SCs are discussed briefly. Perovskite SCs have two major structures: 3D mesoporous and 2D planar. Mesoporous structure typically comprises glass/TCO/c-TiO_2_/m-TiO_2_/perovskite/HTL/metal electrode. The c-TiO_2_ is a compact ultrathin layer of TiO_2_, playing the role of electron transport layer (ETL), whereas hole transport layer (HTL) is a material that has the ability to transport holes only, such as spiro-OMeTAD (2,2′,7,7′-Tetrakis [*N*,*N*-di(4-methoxyphenyl) amino]-9,9′-spirobifluorene). The m-TiO_2_ is a mesoporous structure made of TiO_2_ nanoparticles which serves as a scaffold or platform for the growth of perovskite crystals. The transparent conductive oxide (TCO) is a transparent metal oxide, such as fluorine-doped tin oxide (FTO), sputtered on glass. The planar configuration excludes m-TiO_2_, where the perovskite crystals are directly grown on the c-TiO_2_ layer. Figure 7a shows the structure of a mesoporous perovskite SC. The inverted planar structure is the third device architecture in perovskite SCs. This structure (Fig. 7b) is similar to an inverted polymer SC, where the cathode and anode and therefore ETL and HTL are switched. It typically comprises glass/TCO/PEDOT:PSS/perovskite/PCBM/metal electrode. In this case, PEDOT:PSS (poly (3,4-ethylenedioxythiophene) polystyrene sulfonate) or other suitable compounds are used as the HTL, whereas PCBM (phenyl-C61-butyric acid methyl ester) and other suitable electron-transporting materials are used as the ETL. In all configurations, except for the TCO and metal electrodes that are usually deposited by thermal evaporation or sputtering, other layers are solution-processed. The heart of this cell, i.e., the perovskite layer, has the formulation of methylammonium lead halide, CH_3_NH_3_PbX_3_, where *X* = I, Br, or Cl, a halogen or a combination of several halogens. The aforementioned perovskite molecule is an organic–inorganic compound, made by the reaction of PbX_2_ and CH_3_NH_3_X dissolved in proper solvents. The precursor solutions are deposited by a casting method, in one or two steps. Perovskite may be deposited using the evaporation of precursors, as well. The compact and mesoporous TiO_2_ layers are usually deposited by spray pyrolysis, which is a scalable method [52]. Besides spin coating, scalable methods, such as substrate vibration-assisted drop-casting [6], spray coating [14, 57, 58], blade coating [59], and slot-die coating [60], have been employed to fabricate the perovskite layer, as well. However, the complex nature of the perovskite solution precursors, which makes them easily susceptible to unwanted crystallization, makes the deposition process by a scalable method quite challenging. Also, after deposition, the formation of rather large perovskite crystals usually leads to dewetting due to crystallization [61], resulting in low perovskite coverage. The above-mentioned difficulties make the fabrication process of perovskite layer hard to control and therefore non-reproducible. However, the major challenge the perovskite SCs are facing now is the instability of the cell and the need for well-controlled environment for device fabrication and testing. Therefore, it seems that the research in perovskite SCs is at a critical point now. If the stability issue cannot be addressed within few years, the hope for commercialization of this high-efficiency technology may fade away.

**Fig. 8:**
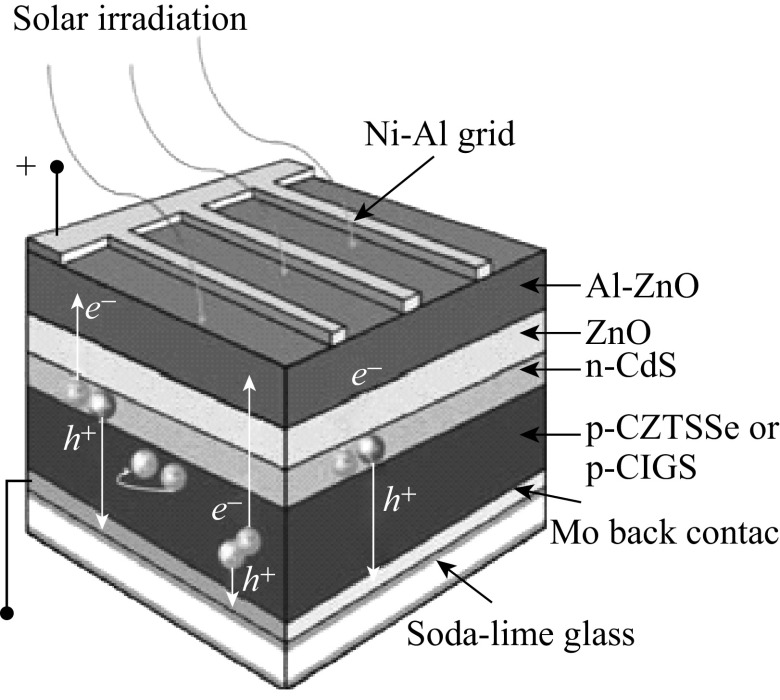
Structure of thin film chalcopyrite (CIGS) and kesterite (CZTSSe) solar cells

Thin film chalcopyrite SCs, based on Cu(In,Ga)Se_2_ (CIGS) and related alloys, are usually deposited using vacuum-based vapor phase methods, but can be processed in solution and casted, as well, to reduce the cost. The vacuum-based CIGS SCs have demonstrated a record PCE of 22.3 % [54]. Figure 8 shows the structure of a CIGS thin film SC. A major concern in CIGS photovoltaics is the scarcity of indium that could potentiality limit the large-scale fabrication of CIGS cells [62]. Using the same device structure, the CIGS absorber layer can be replaced by indium-free absorbers, such as Cu_2_ZnSn(S,Se)_4_ (CZTSSe) with kesterite structure. CIGS and also CdTe SCs are the only thin film SCs that have been commercialized. However, in recent years, some of the main manufacturers of CIGS SCs, such as Solyndra and Nanosolar, went bankrupt, simply because CIGS SCs could not compete with silicon SCs manufactured in China, after a significant drop in the cost of silicon wafers in 2009. Nevertheless, the CIGS SCs are under further development, with the hope to achieve silicon-like efficiencies, while maintaining the manufacturing costs low by developing solution-processed fabrication methods.

Kesterite SCs, utilizing Cu_2_ZnSnS_4_ (CZTS), Cu_2_ZnSnSe_4_ (CZTSe), and Cu_2_ZnSn(S,Se)_4_ (CZTSSe), are promising replacement for the chalcopyrite CIGS absorbers, through the substitution of indium with comparatively abundant and lower cost zinc and tin (Fig. 8). Kesterite SCs are currently less efficient than chalcopyrite SCs and have reached a PCE of 12.6 % [63]. Nevertheless, kesterite light absorbers have a high absorption coefficient and a direct band gap in the range of 1.0–1.5 eV, making them a good choice for photon harvesting in thin film inorganic devices. Although the kesterite light absorbers are inorganic, the cells made by solution processing are even more efficient than those made by vacuum-based methods. The physical limit of PCE of CZTSSe kesterite cell, known as the Shockley–Queisser (SQ) limit, is 31 % under terrestrial conditions [62], much higher than the highest achieved PCE reported so far. Therefore, there is a significant potential for improvement in kesterite SCs, through better control and engineering of layers and interfaces.

The most common vacuum-based process to deposit chalcopyrite and also kesterite layers is by co-evaporation of the metal constituents at room temperature, followed by annealing the resulting film in the presence of selenide vapor. An alternative process is to co-evaporate all elements onto a heated substrate. This process is successful in terms of the PCE of the cell, but imposes excessive costs associated with the vacuum and evaporation processes. As mentioned above, both chalcopyrite and kesterite thin film SCs have been also fabricated using solution-processed and scalable casting methods. For the fabrication of the absorber layer, two processes, either sequentially or in one step, are required: a chemical route to prepare the inorganic absorber material from some cheap precursor solutions and a casting step to deposit a layer of the absorber. In the sequential method, an ink may be prepared by mixing proper solvents with synthesized colloidal nanoparticles of the light absorber. The nanoparticles may be synthesized using a standard wet chemistry or gas-phase method, such as sol–gel, solvothermal, microwave-assisted, etc. The ink then may be deposited by spin coating [64], inkjet printing [65–67], doctor blading [68, 69], spray deposition [70–72], electrophoresis [73], etc. Electrophoresis is different from the casting methods and works based on deposition of single-charged colloidal particles (not ions) onto a charged substrate immersed in the solution. Application of post-heat treatment will result in sintering of nanoparticles and the formation of a smooth film with desired nanostructure and grain boundaries. Alternatively, the synthesis of the light absorber material and the casting may proceed concurrently and in one step, such as that in spray pyrolysis [74–78] and electrospray pyrolysis [79], in which a mixture of the precursor solutions is sprayed onto a hot substrate forming a wet film, where the solution undergoes a chemical reaction (pyrolysis), forming the final thin solid film and some volatile gases. Spray pyrolysis can be used for the fabrication of the buffer layers, such as CdS, as well. Electrodeposition is another one-step method in which the ions of the absorber deposit on the substrate forming the absorber layer [80]. The forgoing discussion reveals the high potential of scalable solution-processed methods for the fabrication of stable inorganic chalcopyrite and kesterite SCs.

**Fig. 9:**
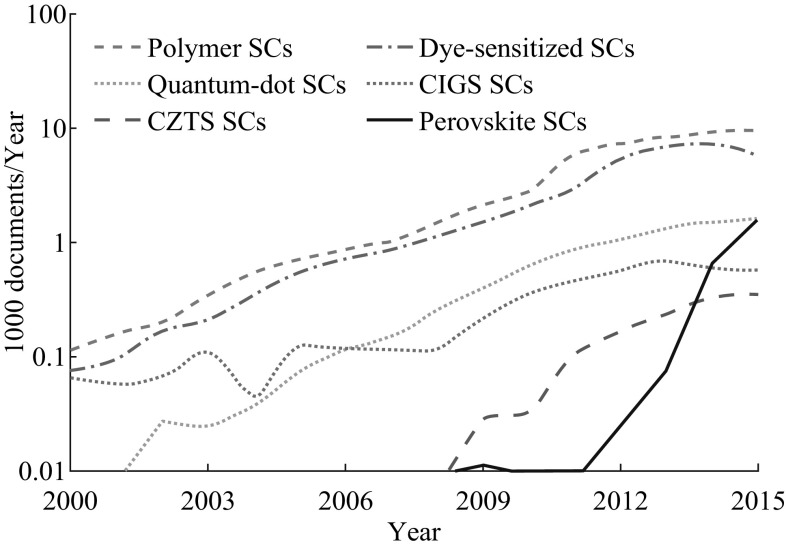
Number of papers found in the Web of Science from 2000 to 2015 on solar cells that can be processed from solution, including polymer, dye-sensitized, quantum dot, perovskite, CIGS, and CZTS SCs

To conclude this section, the number and trend of research papers published on emerging and inorganic CIGS and CZTS SCs in the past 15 years is shown in Fig. 9 . The highest numbers of papers are still published on polymer and dye-sensitized SCs, followed by quantum dot and perovskite SCs. All curves however are concave down indicating a slow-down in research in thin film SCs, after decades of vigorous academic and industry research in this area. An interesting observation is the rapid increase in publications on perovskite SCs within few years, but then reaching the inflection point of the curve in 2014, indicating that the number of publications in this field will continue to increase, but at a slower rate. By revealing inherent deficiencies in perovskite structure, such as the poor stability at room temperature, it is possible that other promising branches of thin film photovoltaics, such as solution-processed chalcopyrite and kesterite SCs, gain ground.

## Thin Film Thermoelectric Devices

**Fig. 10:**
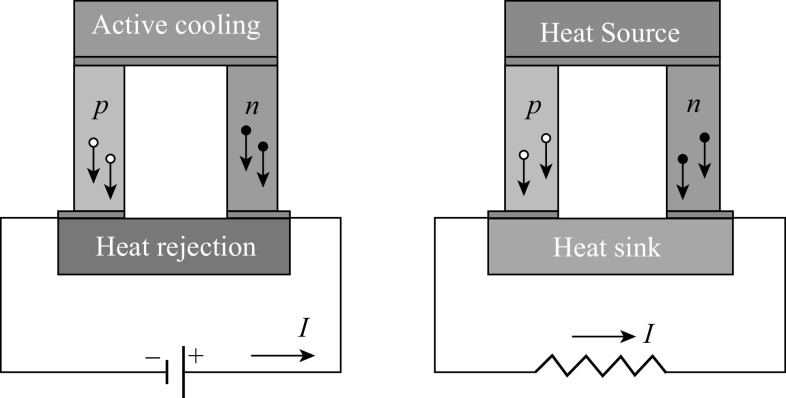
Power generation and cooling modes of a thermoelectric device

In thermodynamics, a heat engine is a device which works between a heat source and a heat sink, and generates mechanical work and electricity (e.g., as in a thermal power plant). A heat pump is the opposite, in that it receives mechanical work to pump heat from a low-temperature reservoir to a high-temperature reservoir (for refrigeration/cooling or heating purposes). Thermoelectric devices are the solid-state version of heat pump and heat engine, operating based on the thermoelectric or the Seebeck–Peltier effect. In the heat pump mode (Peltier effect), when an electric current is driven through a circuit containing two dissimilar materials (ideally an n-type and a p-type semiconductor to boost the effect), heat is absorbed at one junction (the cold side) and released at the other junction (the hot side). This effect results in heat transfer from the cold side to the hot side, thus the device can be used for cooling electronic circuits. In the power generation mode (Seebeck effect), similar to heat engine in thermodynamics, a temperature difference between the hot and the cold sides of the device creates a voltage between the two legs of the device. Therefore, a thermoelectric device may function as either cooling/heating or power generation (Fig. 10). In other words, thermoelectric devices directly convert heat transfer to electricity and vice versa.

The performance of thermoelectric materials is determined by a dimensionless figure of merit *ZT* = *S* ^*2*^ *σT/k*, where *S*, *σ*, *k*, and *T* are the Seebeck coefficient, electrical conductivity, thermal conductivity, and the absolute temperature, respectively. In bulk thermoelectric materials, the typical *ZT* value at room temperature is about 1, although there is no theoretical limit on *ZT*, and higher values may be achieved. For instance, at 300 K, *ZT* in bulk thermoelectric p-type alloy (Bi_2_Te_3_)_0.25_(Sb_2_Te_3_)_0.72_(Sb_2_Se_3_)_0.03_ is 1.14 [81].

The development of thin film thermoelectric devices is in line with ongoing miniaturization of electronic circuits. In addition, thin film thermoelectric devices exhibit faster response time and higher power density compared to bulk devices, making them suitable for localized and small-scale cooling or power generation. Also, owing to unique structural characteristics of thin films, higher values of *ZT* may be achieved in well-controlled thin film thermoelectric materials. This is because, compared to bulk materials, a decrease in thermal conductivity of thin films is expected due to phonon scattering at the film interfaces, and grain boundaries if the film is nanostructured, while the electrical conductivity remains similar to that of the bulk state, resulting in an increase in *ZT*. Venkata et al. [81] measured a *ZT* value of 2.4 in p-type Bi_2_Te_3_/Sb_2_Te_3_ and 1.4 in n-type Bi_2_Te_3_/Bi_2_Te_2_._83_Se_0.17_ superlattices, at 300 K, higher than those realized in bulk thermoelectric materials. Currently, the methods available for the fabrication of thin film thermoelectric devices include energy-intensive evaporation, pulsed laser deposition, molecular beam epitaxy, and magnetron sputtering, limiting their practical widespread applications. Although solution-processed methods are thought to be less suitable for deposition of inorganic materials with proper and defect-free nanostructures, attempts have been made to fabricate inorganic thermoelectric devices using solution-processed methods, just similar to other thin films devices discussed in previous sections. For instance, pre-synthesized nanoparticles of p-type Sb_1.5_Bi_0.5_Te_3_ and n-type Bi_2_Te_2.7_Se_0.3_ alloys were used to prepare stable colloidal solutions or inks for inkjet printing of all-solution-processed flexible thermoelectric device, incorporating solution-processed silver nanoparticles, as the electrode [9]. Balow et al. [82] fabricated Cu_3_(As,Sb)Se_4_ nanocrystalline alloy thermoelectric thin films possessed in solution and deposited by drop-casting. It was argued that the existence of nanoparticles, nanocrystals, and grain boundaries in the film can further result in the scattering of phonons and thus a decrease in thermal conductivity and an increase in the thermoelectric power.

Most currently used thin film thermoelectric materials are inorganic semiconductors, such as Bi_2_Te_3_, PbTe, clathrates, half-Heusler alloys, pentatellurides, and skutterudites [8]. To diversify the potential applications of thin film thermoelectric devices, emerging organic materials with high thermoelectric performance may be alternatively employed. Organic semiconductors have the advantages of variety, low-cost synthesis from solution, mechanical flexibility and transparency, and compatibility with scalable fabrication techniques, although organic materials are usually less stable and the resulting devices are less reproducible compared to their inorganic counterparts. Also, there is a complex relationship between the nanostructure and charge transfer in organic materials, which needs to be understood and controlled. The thermal conductivity of conducting organic materials is typically lower than that of the inorganic materials, which may result in higher *ZT* values. The majority of organic thermoelectric materials studied in the literature are based on p-conjugated polymers (or conducting polymers), such as polyaniline, polyacetylene, polypyrrole, polythiophene, polyphenylene, and PEDOT:PSS, as well as small molecules, such as pentacene, fullerene, and tetrathiafulvalene (TTF), to name a few [83]. The highest *ZT* value in organic thermoelectric materials is 0.42 for spun-on doped PEDOT:PSS co-polymer thin films [84]. PEDOT:PSS thin films as a thermoelectric material have been used by others, as well [85], where the effect of the device geometry on the Seebeck coefficient was studied. As another example of organic materials with thermoelectric capability, 1,1,2,2-ethenetetrathiolate (ett)–metal coordination polymers poly[A_*x*_(M-ett)] (A = Na, K; M = Ni, Cu) possess thermoelectric properties. The *ZT* value of n-type poly[*K*_*x*_(Ni-ett)] can reach 0.2 and *ZT* value of p-type poly[Cu_*x*_(Cu-ett)] can reach 0.01 at 400 K [8]. Jiao et al. [8] used inkjet printing to fabricate a thin film thermoelectric device with several interdigitated p–n legs, based on the aforementioned polymers and gold electrodes. Zhang et al. [86] used a roll-to-roll process for printing flexible thin film thermoelectric devices. PEDOT:PSS inks were used for printing p-type and connection strips and nitrogen-doped graphene dispersed in solvent served as n-type strips. A review of recent works, characterization techniques, optimization procedures, and discussion of theoretical and experimental research paths in thermoelectrics may be found in Ref. [83].

The research directions in this field include the synthesis, optimization, and engineering of high-performance stable inorganic, organic, and inorganic–organic thermoelectric materials, as well as developing low-cost materials with acceptable performance that can be readily processed and deposited from solution. Some examples of recently developed or modified thermoelectric materials include the solution-processed electrically conducting metal–organic framework, tetracyanoquinodimethane (TCNQ) @ Cu_3_(BTC)_2_ thin films [87], thin films of coordination polymer formed by ethylenetetrathiolate and nickel, deposited electrochemically [88], low-cost n-type Al-doped ZnO deposited by sputtering [89] and spray pyrolysis [90], introducing small molecule polyethyleneimine into existing p-type carbon nanotube films by spray coating, and converting it to an n-type composite film for low-cost p–n legs of a spray-on thermoelectric device [12].

Photothermal materials, such as emerging organic materials that have the capability to absorb heat near infrared region, have niche applications, such as in dark-field security cameras and night vision sensors [91]. The light-driven heat could be converted into electricity when photothermal conversion is coupled with the thermoelectric effect, allowing photothermoelectric conversion. Therefore, while in a photovoltaic SC, electricity is generated directly through the photovoltaic effect, in a photothermoelectric device, light is first converted to heat and then the heat transfer generates electricity through the Seebeck or thermoelectric effect. This is important because tailored photothermal materials can absorb electromagnetic energies even outside of the visible range, while most photovoltaic materials do not have this capability.

## Thin Film Gas and Biosensors

Sensors or detectors are devices used to detect and measure the intensity of foreign species, such as toxic or flammable gases in the workplace, exhaled gases in human breath for disease diagnosis, e.g., formaldehyde, carbon dioxide, carbon monoxide, hydrogen, hydrogen sulfide, ammonia, nitric oxide, volatile organic gases, also humidity, ultraviolet or infrared light, stress and strain, biological species, etc. The sensors operate based on responding to the analyte by generating a signal amenable to further processing. The sensing nature may be physical, such as in surface wave acoustic, optical, and infrared sensors, or chemical, such as that in the so-called chemiresistors. Thin films have large surface areas making them one of the best geometries for sensing purposes. The area of thin film sensors is quite large and active, with about 10,000 papers published per year as shown in Fig. 1. Therefore, this section is limited to some research directions in thin film chemiresistive sensors for detection of gaseous and biological targets, to highlight similarities between these devices and other thin film devices, discussed in this paper. A chemiresistor is a material that changes its electrical resistance in response to changes in the nearby chemical environment. For detection of various gases and pollutants or biomaterials in a solution, a specific type of sensor structure and material may be more suitable. Typical chemical sensing materials include metal oxide semiconductors, conducting polymers, and emerging materials, such as graphene and carbon nanotubes, as well as composite materials.

**Fig. 11:**
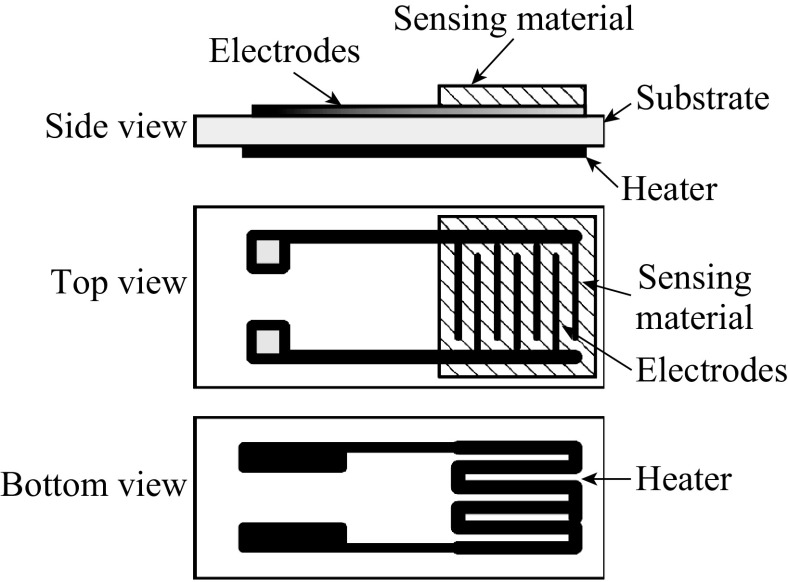
Typical structure of a two-terminal resistance-based (chemiresistive) thin film oxide sensor [93]

Most chemical gas sensing materials are based on metal oxides, such as MoO_3_, SnO_2_, WO_3_, and ZnO, which work based on the following proposed mechanism [92]: In air, typically, oxygen molecules are adsorbed on the surface of the metal oxide, capturing its free electrons. Therefore, this trapping of negative charges of oxygen of the metal oxide causes increased resistance. When the metal oxide is exposed to a reducing target gas, the electrons trapped by oxygen will return to the oxide, leading to a decrease in the metal oxide resistance. Thus, metal oxide chemiresistive sensors detect the presence of a particular gas based on a change in their resistance. Figure 11 shows the structure of a two-terminal resistance-based metal oxide sensor with a heater to optimize the sensitivity [93]. In addition to the conventional two-terminal thin film sensors, which work based on a change in their resistance or conductivity when exposed to a target gas, three-terminal field-effect transistor (FET) is a more accurate measurement option [94]. This is because in a FET several measurable quantities, such as the bulk conductivity of the conducting channel, field-induced conductivity, transistor threshold voltage, and the field-effect mobility of the transistor, may change when exposed to chemical species, allowing for recognition of the molecular species [95]. Such thin film transistor gas sensors are called chemical field-effect transistors (ChemFETs) [94].

**Fig. 12:**
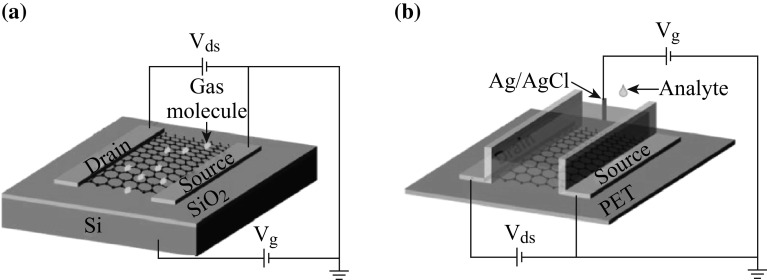
**a** Typical back-gate graphene-based FET on Si/SiO_2_ substrate used as a gas sensor. **b** Typical solution-gate graphene-based FET on flexible polyethylene terephthalate (PET) substrate used as a chemical and biological sensor in aqueous solutions [105]

Similar to other thin film devices, organic semiconductors, such as conducting polymers, are emerging alternatives to inorganic metal oxide semiconductors in chemiresistive thin film sensors. The conductivity of conducting polymers, such as polyaniline, polypyrrole, polythiophene, polyacetylene, and poly (3,4-ethylenedioxythiophene) (PEDOT), is due to the delocalization of *π*-bonded electrons over the polymeric backbone. These polymers show electronic properties, such as low ionization potentials and high electron affinities [96]. Upon exposure to the analyte gas, the conducting polymers are usually doped by redox reaction leading to an increase in their conductivity [97], thereby providing impedance (resistance) type chemiresistive sensors [98, 99]. Conducting polymers may be mixed with highly conductive materials, such as graphene, carbon black, carbon nanotubes [100], and nanoparticles [101], to form composite sensing materials. In such composite sensors, the conducting additives serve as the conduction paths, while the polymer provides selectivity to the gas or biological analytes [102, 103]. Creatinine, cholesterol, glucose, urea, amino acids, protein, and DNA are some examples of biological analytes. The porosity of composite sensing materials can be tuned to increase the sensitivity of the material. Conducting polymers are organic and therefore more versatile in terms of their characteristics, and are more sensitive but less stable and durable compared to their inorganic counterparts [104]. Figure 12 compares a typical gas sensing system using a graphene-based field-effect transistor, in which the channel is directly exposed to the target gas, with a similar device used in the liquid solution. For liquid-phase sensing, the graphene channel is immersed in the sensing chamber, and the drain and source electrodes are insulated to prevent current leakage from the ionic conduction. The gate, usually Ag/AgCl or Pt, is immersed in the solution. The sensing mechanism may be due to the electrostatic gating effect (the charged molecules adsorbed on graphene act as an additional gating capacitance altering the conductance of the graphene channel), the doping effect (similar to gas sensors), or a combination of the aforementioned effects [105]. Graphene thin films may be deposited by solution-based methods, such as spray coating [10], which is preferred over vacuum-based methods, such as CVD. A review of various types of biosensors based on organic thin film transistors for detection of pH, ion, glucose, DNA, enzyme, antibody–antigen, and dopamine may be found in Ref. [106].

Conducting polymer thin films may be deposited by various solution-processed casting techniques, such as electrochemical deposition, Langmuir–Blodgett (LB) technique, layer-by-layer self-assembly, dip coating, spin coating, drop-casting, spray coating, inkjet printing, as well as various forms of thermal evaporation at moderate temperatures [97]. Compared to organic materials for sensors, inorganic semiconductor oxides are durable, stable, and resistant to harsh environmental conditions. Various evaporation-based and solution-based techniques may be used to deposit inorganic thin film sensors. Organic materials are usually dissolved in a solution and deposited based on a single-step physical process. In metal oxides, however, solution-processed casting techniques may be accompanied by a chemical process, such as sol–gel (e.g., sol–gel followed by spin or dip coating) or pyrolysis (e.g., spray pyrolysis) to develop metal oxides from their precursor solutions. Development of scalable casting methods is of importance for paving the way for commercialization of such technologies. Spray technology as a scalable technique may be utilized to deposit both organic sensors (spray deposition or spray coating) and also inorganic sensors (spray pyrolysis), where spray pyrolysis is performed at elevated temperatures and is accompanied by a chemical reaction to form inorganic ceramics. To fabricate ammonia sensors, Mani and Rayappan [107] deposited nanostructured ZnO thin films on glass substrates using spray pyrolysis followed by annealing. In another study, Xie et al. [108] fabricated ChemFET ammonia gas sensors using poly (3-hexylthiophene) (P3HT)/molybdenum disulfide (MoS_2_) composite organic/inorganic thin film by spray coating, where a short recovery sensing time was achieved with a change in ammonia concentration. Zhou et al. [100] fabricated spray-on gas sensors made of conducting polymer polyethylene oxide and carbon nanotubes (both mixed composite film and bilayer stacked films) to improve the film conductivity. The sensor was tested for detection of toluene vapor, where the bilayer device showed higher sensitivity and selectivity to toluene. Tai et al. [101] developed a formaldehyde ChemFET sensor based on P3HT (poly-3-hexylithiophene)/Fe_2_O_3_ nanocomposite thin film by spray coating, in which the channel length and width were as small as 25 and 4000 μm, respectively. The improved performance of the composite sensor was attributed to the porous surface of the composite film. For deposition of such small-area thin films by spray coating, masks need to be employed. Therefore, for such small areas, alternative scalable methods, such as inkjet printing or electrosprays, may be preferred to minimize material wastage.

In a study [109], polycrystalline ZnO-based thin films doped with Cu were deposited on glass substrates, using spray pyrolysis to fabricate H_2_S gas sensor. Pandeeswari and Jeyaprakash [110] used spray pyrolysis to deposit polycrystalline gallium oxide thin films onto quartz substrates with gallium acetylacetonate as the precursor salt, to fabricate ammonia sensors. Solution precursor plasma spraying is another scalable technique for the synthesis and casting of oxide sensors. Zhang et al. [111] deposited ZnO nanostructured sensing coatings using this method, with zinc acetate as the precursor solution. The high temperature of plasma essentially vaporizes the entire precursor solution (solvent and solute), such that the film forms from the condensation of vapor of species, thus the method is different from spray pyrolysis, which is a droplet-to-particle spray method [112]. In fact, the solution precursor plasma spray is similar to flame spray pyrolysis [113], in which the precursor solution is converted to vapor in a burning spray. The method is suitable for the fabrication of composite ceramic particles and thin films. Electrosprays are generally more controllable than conventional sprays and may be more suitable for fabrication of small-area thin films. Jamal et al. [20] fabricated nickel oxide hydrogen sensors by electrospray pyrolysis deposition, using hydrated nickel chloride as the precursor solution. Inkjet printing is also a successful scalable method for the fabrication of small-area thin film sensors [114–117]. A proper ink has to be prepared for this purpose, similar to other thin film devices fabricated by inkjet printing. The ink may be a colloidal solution of previously synthesized nanoparticles or a solution of the precursor solutions that would undergo a chemical reaction in situ, such as that in sol–gel, to produce the sensing material in the form of a thin film, after the required heat treatment and annealing.

## Thin Film Smart Materials and Actuators

A smart material has the intrinsic capability of responding to external stimuli, such as stress, temperature, moisture, pH, and electric or magnetic fields, in a predictable and controllable manner, in an appropriate time, and ideally returns to its original shape, as soon as the stimuli are removed. SMMs and electromechanical materials (e.g., piezoelectric ceramics) are examples of smart materials, widely used as physical sensors and actuators [118]. These materials can be integrated with conventional silicon-based micro-electro-mechanical systems (MEMS) and other thin film mechanical devices to develop new applications.

The SMMs can generate motion or store energy desirable for various applications, such as automotive, aerospace, robotics, and biomedical. SMMs are usually in the form of shape memory alloys (SMAs), such as copper–aluminum–nickel and nickel–titanium alloys in the bulk form, but alternative materials, such as ceramics and polymers, and alternative shapes, such as thin films and fibers, are emerging. SMMs may be patterned by lithography on deposited thin films and used in MEMS, as various types of actuators and sensors, such as micro-switches, micro-relays, micro-pumps, micro-valves, micro-grippers, and micro-positioners. Shape memory polymers (SMPs) are emerging SMMs, which are soft, organic, and biodegradable, and suitable for biomedical, textile, and surface patterning, to name a few [119, 120]. One drawback of SMPs is that despite enormous increase in knowledge about their design principles, revealing two-way shape memory effect (SME) in SMPs is still rare [121]. In a two-way SME, the polymer should return to its original shape, as soon as the stimulus is removed. Reports on the fabrication of thin film SMPs are rare, given that this is an emerging although promising research area. Lei et al. [122] fabricated hybrid polystyrene-based nanocomposite SMP thin films with skeletal structures of carbon nanotubes formed inside, using solution-processed casting methods. With further development, it is expected that new solution-processed thin film SMPs will emerge in the future.

**Fig. 13:**
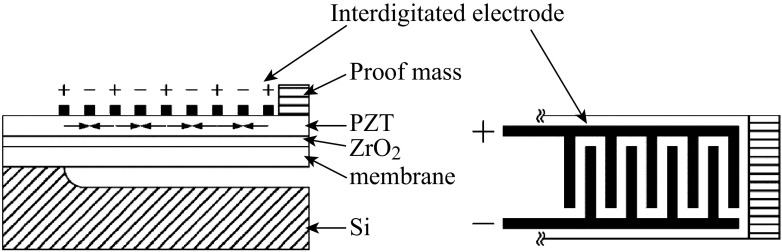
Piezoelectric thin film device for generation of electricity from mechanical vibration. The silicon oxide is deposited via chemical vapor deposition, whereas the ZrO_2_ and PZT layers are processed and deposited from solution [126]

Another important family of smart materials is piezoelectric materials, such as poled polycrystalline ceramics (e.g., lead zirconate titanate, PZT), single-crystalline or highly oriented polycrystalline ceramics (e.g., zinc oxide and quartz), organic crystals (e.g., ammonium dihydrogen phosphate), and polymers (e.g., polyvinylidene fluoride) [123]. A piezoelectric material is a material that develops a dielectric displacement in response to an applied stress and, conversely, develops a strain in response to an electric field. The piezoelectric effect can be used in sensors, actuators, and transducers, where their pyroelectric effect (generation of a temporary voltage when heated or cooled) can be used in infrared motion sensors [124]. In addition to the aforementioned traditional applications, piezoelectric energy-harvesting devices have been developed to harvest electrical energy in micro- and nanoscale from ambient mechanical energies created by natural sources or from human movements [125, 126]. Figure 13 shows a solution-processed thin film piezoelectric microcantilever device working at its resonance mode for utilizing environmental vibration for micro-power generation, as well as monitoring environmental vibration [126]. The PZT layer was processed in sol–gel condition and deposited by spin coating.

**Fig. 14:**
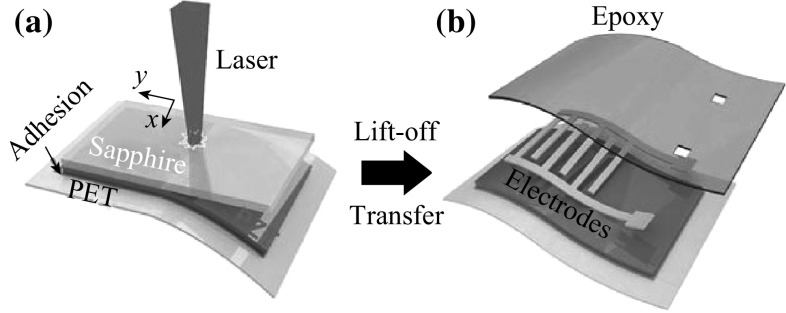
Schematic diagram of the fabrication process of a solution-processed thin film PZT energy-harvesting device, using environmental micro-vibration as the source of mechanical energy. Using laser, a 2-μm spun-on PZT thin film deposited on transparent sapphire (**a**) was transferred onto a flexible PET plastic substrate (**b**), where electrodes and protective layer were deposited to complete the device [125]

Among the above-mentioned piezoelectric materials, some of them, for example lead zirconate titanate (PZT), have ferroelectric property, as well. Ferroelectric materials have an additional restriction in their crystal structure, which is to possess a direction of spontaneous polarization. This inherent polarization can be oriented by the application of an electric field which will be maintained to some degree when the field is removed [123]. The PZT is a solid solution of ferroelectric PbTiO_3_ and anti-ferroelectric PbZrO_3_ and has a perovskite ABX_3_ structure. Deposition and growth of PZT thin films, as the most widely used piezoelectric materials, have been achieved by physical and chemical evaporation-based routes, such as sputtering and chemical vapor deposition, as well as solution-based wet chemistry methods. Physical and chemical vapor deposition routes are well studied and generally produce uniform and intact films. However, controlling the correct stoichiometry in these methods is challenging. Solution-processed methods are cheap and provide better control over the stoichiometry of the thin film, although obtaining uniform and intact films during the casting step is challenging. As mentioned earlier, the solution-processed methods consist of several sequential or combined steps including a possible chemical reaction step, such as sol–gel, a film deposition step, such as spin coating, and post-heat treatment to remove residual bonds and volatiles. All of the aforementioned steps are crucial for obtaining an intact and uniform film with right stoichiometry and crystal structure. As an example, using solution-processed methods, nanoscale PZT energy generators were fabricated on flexible substrates [125]. A piezoelectric inorganic material was synthesized by a conventional sol–gel method, and then the solution was spin-casted on a double-sided polished sapphire substrate. Heat treatment was performed to crystallize the as-casted amorphous film. A lift-off laser technique was used to transfer the piezoelectric thin film from bulk sapphire substrates to plastic substrate. The device was completed by deposition of electrodes and encapsulation by epoxy as depicted in Fig. 14. This device has the potential to be made by scalable casting techniques.

Besides inorganic ferroelectric and piezoelectric materials, recent advances made in polymer science have resulted in the emergence of smart organic materials, such as electroactive, conjugated, and polymer–metal composites, with similar traits as those of their inorganic counterparts. Electroactive ionic and dielectric polymers respond to electrical stimulation by large changes in size and shape, making them suitable for use in sensors, actuators, and soft robots. These soft yet robust polymer materials typically provide larger strains than conventional piezoelectric materials. These polymers may be used in various forms, including thin films, to fabricate actuators, such as dielectric elastomers [127]. In a dielectric elastomer, a dielectric electroactive polymer thin film is sandwiched between two electrodes. On activation, a large electric field is applied on the electrodes; therefore, the charged electrodes are attracted toward each other, compressing the dielectric film and causing a decrease in dielectric film thickness and an increase in the film area. Thus, an electric field may result in a reversible mechanical force and motion. Conjugated conducting polymers [128] and ionic polymer–metal composites [129] are other smart materials that can be used to design soft actuators. They present large actuation amplitudes while requiring low voltages. Although the energetic efficiency of such actuators is low in comparison to ceramic piezoelectric actuators, they have a larger displacement. These polymeric thin film devices may be processed in solution at lower temperatures compared to their inorganic counterparts, and can be readily deposited using a low-cost casting method. Inorganic ferroelectric and piezoelectric materials usually require higher temperatures to obtain the required crystalline phase. This high-temperature treatment limits the deposition of such materials on plastic substrates. This could be overcome by using organic ferroelectrics that display much lower processing temperatures. However, given that inorganic materials are robust, stable, reproducible, and reliable, developing low-temperature processing methods for inorganic ferroelectric thin films for the purpose of scalability and compatibility with plastic substrates is desirable. The use of UV irradiation lowers the process temperature of inorganic materials. Also the development of seeded diphasic sol–gel precursors can result in an appreciable reduction in crystallization temperature of ferroelectric oxides. The combination of the above-mentioned strategies was used by Bretos et al. [130], where PZT thin films were spun on flexible polyimide substrate. In another work [131], a ferroelectric-gate thin film transistor memory was fabricated, where PZT film was used as the capacitor, processed in sol–gel environment, and deposited on glass substrates. It was found that the PZT crystallinity is improved at higher annealing temperature, but the transistor leakage is better if PZT is annealed at a lower temperature (500 °C). Therefore, high crystallinity may not be the main requirement for an inorganic thin film, as also observed in kesterite SCs discussed before [62].

Each of the various electromechanical actuating materials discussed above (ferroelectric/piezoelectric ceramics, electroactive polymers, and conjugated conducting polymers) excel in some aspects, but may have unsatisfactory performance in other aspects. Ferroelectric ceramics show high energy density, but small actuation strain, electroactive polymers exhibit large actuation deformation and strain, but require very high activation voltage, and conjugated polymers require low activating voltage, but suffer from short response rate. Using composite and nanocomposite materials may alleviate some of the shortcomings mentioned above. Liang et al. [132] employed polymer polydiacetylene (PDA) materials and flexible graphene paper and fabricated solution-processed graphene/PDA bimorph actuators, demonstrating controllable and partially reversible bending motion with fast response, activated by a low voltage. The improved actuation performance was attributed to the effective combination of the features of graphene and electrically and thermally induced expansion mechanism of the PDA crystal.

## Summary and Conclusions

In this paper, principles of operation, characteristics, and fabrication process of some of the most popular, widely investigated, and promising thin film devices, including this film transistors, light-emitting diodes, solar cells (SCs), thermoelectric devices, sensors, actuators, and smart materials, were critically reviewed. Some of the advances made in emerging thin film devices are indebted to the development of functional and smart materials, with organic, inorganic, and composite nature or structure. These include but are not limited to ferroelectric/piezoelectric ceramics, shape memory materials (SMMs), electroactive polymers, conjugated conducting polymers, and molecular semiconductors. The discovery and synthesis of carbon nanotubes, graphene, and other nanomaterials has resulted in further improvement of characteristics of the advanced materials. Owing to the similarity in operation and multidisciplinary nature of thin film devices, advances made in one area may be used in other similar areas. For instance, molecular semiconductors, such as conducting and semiconducting polymers and perovskites, developed for SCs, may be employed in other devices, such as thin film transistors, sensors, etc. Or carbon nanotubes and graphene may be incorporated in most thin films to improve the film characteristics and the device performance.

Owing to the advantages of solution-processed thin film deposition methods, such as processing in ambient conditions to reduce the fabrication cost, this work focused on inorganic and organic materials that can be processed in liquid solution and deposited using low-cost scalable methods. It was elaborated that solution-processed deposition methods may be physical or chemical. In physical processing, the precursor materials are dissolved (e.g., organic materials) or dispersed (e.g., colloidal nanoparticles) in proper solvents and liquids and are deposited using a casting method, such as spin coating, drop-casting, inkjet printing, spray deposition, and slot-die coating. Heat treatment may be performed to improve the film nanostructure and vaporize the solvent residues, or to sinter deposited nanoparticles. In chemical solution processing, the precursors undergo a chemical reaction in the solution or after deposition during the heat treatment, such as that in spray pyrolysis, sol–gel, and solvothermal methods. The chemical route is usually applicable to solution processing of inorganic materials, whereas the physical route usually applies to organic materials, such as polymers. It was also argued that in applications where high crystallinity is not essential for device performance, inorganic materials may be processed and deposited from solution, instead of using energy-intensive evaporation route.

It is deduced that synthesizing new organic and inorganic materials with high transparency, charge mobility, and stability and developing reproducible and scalable solution-processed deposition methods are required to pave the way for commercialization of low-cost solution-processed thin film transistors for application in flat-panel display backplane, flexible displays, gas- and biosensors, data storage memories, etc. In the field of thermoelectric devices, the research is focused on developing solution-processed nanostructured thermoelectric materials used as thin films, to achieve higher thermoelectric figure of merit compared to bulk materials, suitable for efficient microscale heating/cooling and energy harvesting. In the area of thin film SCs, the development of inorganic chalcopyrite and kesterite SCs with compelling efficiencies, processed in solution and fabricated using scalable methods, is a promising research direction, to reduce the cost of the delivered energy. This is because the aforementioned inorganic SCs are stable and do not have the difficulties associated with organic and organic–inorganic materials, such as polymers and perovskites. Further effort to resolve the stability issue in perovskite SCs is essential for the future success of this emerging technology.

In this review, the research trends in chemical gas- and biosensors based on two-terminal (resistive) and three-terminal (field-effect transistor) structures were studied, as well. The challenge is to synthesize organic and inorganic sensing materials, ideally solution-processed, with high selectivity, sensitivity, and predicable response to an analyte. Doping of the sensing materials with graphene, carbon nanotubes, and nanoparticles may improve their performance, such as their sensitivity. In the area of smart materials, SMMs and electrochemical materials, such as piezoelectrics, are currently considered for actuation, physical sensing, and energy harvesting in microscale. Thin film actuators may be incorporated into micro-electro-mechanical systems (MEMS) for emerging applications.

The importance of developing scalable casting methods was also highlighted. Some recent works that employed scalable methods, such as inkjet printing, spray coating, and slot-die coating for the fabrication of thin film devices, were reviewed. To substantiate the importance of scalable methods, it was argued that for instance the price could be substantially reduced if OLED displays, currently deposited by evaporation, could be deposited by spray coating and other low-cost methods. The knowledge gained through the development of lab-scale devices may not be easily extendable to large-scale and real-world applications. Therefore, fundamental studies are required to understand and control the process of thin liquid film formation, solvent evaporation, film drying, and the physical and material properties of the resulting thin solid films, such as the film nanostructure.
